# Interleukin-8 as a Biomarker for Disease Prognosis of Coronavirus Disease-2019 Patients

**DOI:** 10.3389/fimmu.2020.602395

**Published:** 2021-01-08

**Authors:** Lili Li, Jie Li, Meiling Gao, Huimin Fan, Yanan Wang, Xin Xu, Chunfeng Chen, Junxiao Liu, Jocelyn Kim, Roghiyh Aliyari, Jicai Zhang, Yujie Jin, Xiaorong Li, Feng Ma, Minxin Shi, Genhong Cheng, Heng Yang

**Affiliations:** ^1^ Center for Systems Medicine, Institute of Basic Medical Sciences, Chinese Academy of Medical Sciences & Peking Union Medical College, Beijing, China; ^2^ Cheng Lab, Suzhou Institute of Systems Medicine, Suzhou, China; ^3^ Department of Laboratory Medicine, TaiHe Hospital, Hubei University of Medicine, Shiyan, China; ^4^ Department of Scientific Research, Suzhou Func Biotech Inc, Suzhou, China; ^5^ Department of Microbiology, Immunology & Molecular Genetics, University of California, Los Angeles, Los Angeles, CA, United States; ^6^ Department of Surgery, Affiliated Tumor Hospital of Nantong University, Nantong, China

**Keywords:** cytokine serum profile, biomarker, coronavirus disease-2019 prognosis, respiratory syndrome coronavirus 2, cytokine storm, interleukin-6, interleukin-8

## Abstract

The widespread prevalence of coronavirus disease-2019 (COVID-19) which is caused by severe respiratory syndrome coronavirus 2 (SARS-CoV-2) infection, has resulted in a severe global public health emergency. However, there are no sensitive biomarkers to predict the disease prognosis of COVID-19 patients. Here, we have identified interleukin-8 (IL-8) as a biomarker candidate to predict different disease severity and prognosis of COVID-19 patients. While serum IL-6 become obviously elevated in severe COVID-19 patients, serum IL-8 was easily detectible in COVID-19 patients with mild syndromes. Furthermore, lL-8 levels correlated better than IL-6 levels with the overall clinical disease scores at different stages of the same COVID-19 patients. Thus, our studies suggest that IL-6 and IL-8 can be respectively used as biomarkers for severe COVID-19 patients and for COVID-19 disease prognosis.

## Introduction

Since the initial outbreak in December of 2019, severe respiratory syndrome coronavirus 2 (SARS-CoV-2) infection and its associated coronavirus disease-2019 (COVID-19) has quickly developed into a global epidemic (1–3), causing over 70 million confirmed COVID-19 cases and 1.6 million associated deaths (2, 4–6). SARS-CoV-2 is a novel betacoronavirus belonging to the large coronavirus family. The coronavirus family includes Alphacoronavirus [i.e. Human coronavirus 229E, Human coronavirus NL63), Betacoronavirus (SARS-CoV, SARS-CoV-2, Middle East respiratory syndrome (MERS), Human coronavirus OC43), Gammacoronavirus (Avian coronavirus (IBV)], and Deltacoronavirus [Bulbul coronavirus HKU11 (Bulbul-CoV HKU11)] (7, 8). In the past two decades, two large scale pandemics have been caused by coronavirus, SARS in 2003 and MERS in 2012 (9, 10). SARS-CoV-2 infection was firstly reported at the end of 2019 and rapidly spread across the world in a few months, posing a major threat to the health of people around the world.

Symptoms of COVID-19 include fever, cough, shortness of breath, tissue damage, multiorgan failure, and death. The outcomes of COVID-19 vary from mild symptoms to acute resolved or fatal pneumonia. Most COVID-19 patients were classified as mild (81%), 14% as severe, and 5% as critical. The overall case-fatality rate (CFR) was 2.3% and all the deaths were from critical patients who represented about almost half of critical cases (11). Elderly patients and those with preexisting co-morbid conditions have higher risks to develop critical illness and die from the COVID-19 disease (11, 12).

Diagnosis for COVID-19 patients includes two main methods, nucleic acid testing for detection of SARS-CoV-2 RNA and serological assay for detection of specific IgG and IgM antibodies against SARS-CoV-2 (13). To avoid the false negative rate of reverse transcriptase polymerase chain reaction (RT-PCR) test and improve the accuracy of diagnosis, researchers have suggested a strategy of combining RT-PCR test, antibody test, and CT scan for COVID-19 diagnosis (14–16). Meanwhile, some researchers also tried to find predictors of COVID-19 disease prognosis. Although the viral load, viremia, and RNA in sera of SARS-CoV-2 correlated with the severe outcome in COVID-19 patients (17–20), sensitive biomarkers representing the prognosis of COVID-19 patients are stilled lacked.

SARS-CoV-2 infection induces exuberant inflammatory responses and increased secretion of interleukin-1β (IL-1β), interferon-γ (IFN-γ), interferon inducible protein 10 (IP-10), monocyte chemotactic protein 1 (MCP-1), interleukin-4 (IL-4), and IL-10. In addition, intensive care unit (ICU) patients with more severe disease process have higher plasma levels of IL-2, IL-7, IL-10, granulocyte colony stimulating factor (GCSF), IP-10, MCP-1, macrophage inflammatory protein-1a (MIP-1A), and tumor necrosis factor-α (TNF-α) than non-ICU patients (21–24). Among severe COVID-19 cases, the majority of the patients with respiratory distress syndrome were associated with high systemic IL-1β, TNF-α, and IL-6 levels (25, 26). These reports suggest a possible connection between proinflammatory cytokine induction and adverse effects of COVID-19. IL-6 is considered a relevant indicator in predicting severe course of COVID-19 disease and target for COVID-19 treatment (27–31). Besides inflammatory cytokines, some other predictors, such as lymphocyte number, lactate dehydrogenase, D-dimer, and IL-2R have also been identified as out of normal range in COVID-19 patients (12, 32, 33). Nevertheless, available biomarkers for predicting the disease progression of COVID-19 patients are still limited.

In this study, we characterized the serum profiles of 40 cytokines in COVID-19 patients at different disease stages. While IL-6 was an available indicator in severe COVID-19 patients, IL-8 performed better in indicating the progress of COVID-19 disease status from mild to severe. IL-8 plasma levels were elevated in both mild and severe COVID-19 patients and increased with disease progression. Our work showed that IL-6 and IL-8 could be combined used as biomarker for different disease stages of COVID-19 patients.

## Materials and Methods

### Quantibody Human Inflammation Array Assay

**Figure 1:**
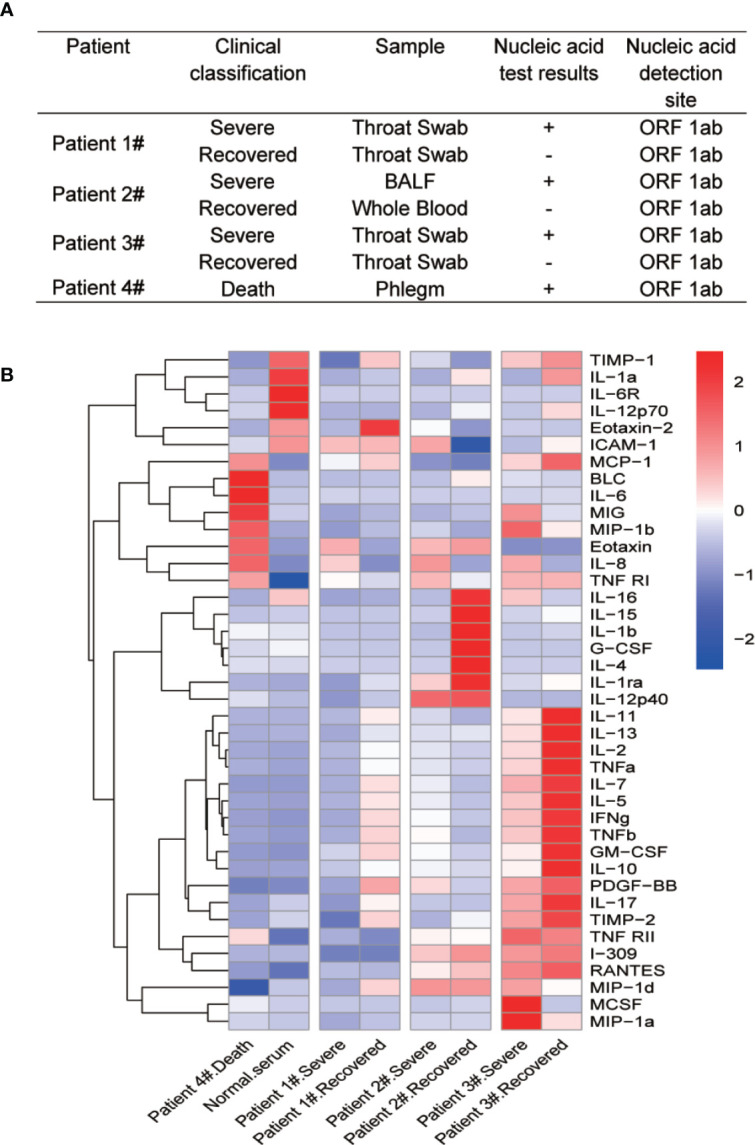
Multiple cytokine profile from different clinical outcomes of SARS-CoV-2 infected patients. **(A)** Clinical outcomes and nucleic acid test results of four SARS-CoV-2 infected patients. The patients were divided into two groups by clinical outcomes: severe and recovered. Patient 4# died in the hospital. **(B)** Heatmap of cytokines in sera from COVID-19 patients during different clinical stages. Because of the death, we did not get recovered serum from patient 4#. We then added normal serum as a control. Cytokine levels were detected using Quantibody^®^ Human Inflammation Array-3.

Forty human cytokines of 8 COVID-19 patients’ sera were evaluated using Quantibody Human Inflammation Array-3 (RayBiotech, Inc.) according to the manufacturer’s protocol. After incubated with 8 COVID-19 patients’ sera and 8 standard samples, the slides were scanned using a laser scanner (Axon GenePix; Molecular Devices) and images were collected with GenePix Pro 6.1.0.4 software (Molecular Devices). Data from the images were further gathered using GenePix Array list files (RayBiotech, Inc.) and converted to concentration with RayBio Q Analyzer software (RayBiotech). The comparative concentrations of the 40 cytokines were displayed with heatmap as indicated in **Figure 1B**.

### Clinical Investigations and Data Analysis

Clinical investigations in COVID-19 patients and healthy volunteers were approved by the Ethics Committee of Taihe hospital (2020KS031). Sera from human donors were collected after the patients were admitted to the hospital with obvious SARS-CoV-2 infection symptoms like fever, radio-graphic evidence of pneumonia, low or normal white-cell count or low lymphocyte count and positive SARS-CoV-2 nucleic acid testing. All the sera were collected under a protocol approved by the local governing human research protection committee and used under the approvement of Taihe hospital. We used a modified Acute Physiology and Chronic Health Evaluation (APACHE II) scoring system to evaluate the disease severity of 5 patients during the hospitalization. Briefly, the APACHE II score of each patient was evaluated based on 12 physiologic measurements (Body temperature, mean arterial pressure, heart rate, respiratory rate, alveolar-arterial oxygen (A-a) gradient; if fractional inspired oxygen concentration is ≥0.5, arterial oxygen tension (PaO^2^); if fractional inspired oxygen concentration is <0.5, serum bicarbonate (HCO_3_); if there is no arterial blood gas analysis, arterial pH, serum sodium, serum potassium, creatinine, hematocrit, white blood cell count, Glasgow Coma Scale score), age of the subject and comorbid conditions. Physiologic signs and laboratory values used to calculate the APACHE II score must have been obtained using the worst physiologic variable within the 24 h period. A higher score is associated with more severe disease and a higher risk of hospital death.

### Enzyme-Linked ImmunoSorbent Assay

IL-6 and IL-8 levels in COVID-19 patients and healthy people’s sera were measured with Human IL-6 ELISA Set (BD biosciences) and Human IL-8 ELISA Set (BD Biosciences) according to the manufacturer’s protocol. Briefly, the plates were coated with the capture antibodies at 4°C for overnight and then washed with 200 μl/well 1xPBST buffer (1xPBS supplemented with 0.05% Tween-20) for 3 times. These coated plates were blocked with 100 μl/well blocking solution (1% BSA in PBS buffer) at 37°C for 1 h. After washing three times, 100 μl/well diluted sera were added into the plates and incubated at 37°C for 2 h, followed by washing for three times. Diluted capture antibodies at 100 μl/well were then added into the plates and incubated at 37°C for 1 h, followed by washing for three times. Diluted secondary antibody was added at 100 μl/well into the plates and incubated at 37°C for 1 h, followed by washing for five times. As the final step of ELISA, 100 μl/well 3,3′,5,5′-Tetramethylbenzidine (TMB) was added into the plate and incubated for 5–10 min with light protection and the reaction was terminated by addition of 50 μl/well 2M H_2_SO_4_. The absorbance at 450 nm in individual wells were measured by SpectraMax^®^ i3 (Molecular Devices) plate reader.

### Statistics

Statistical analyses were performed with GraphPad Prism 7 software and R Studio version 3.6.3. The continuous variables were presented as mean ± SD. Data with normal distribution were analyzed by one-way ANOVA or unpaired two-tailed Student’s *t* tests, and P values were indicated by ns, not significant, * *P* < 0.05, ** *P* < 0.01, *** *P* < 0.001, and **** *P* < 0.0001.

## Results

### Multiple Cytokine Profile From Different Clinical Outcomes of SARS-CoV-2 Infected Patients

Cytokine storm has been found to be a part of COVID-19 associated severe diseases. To identify differential cytokine profiles potentially associated with disease status of COVID-19 patients, we tested 40 human inflammatory related cytokines in the sera of 4 hospitalized patients with confirmed COVID-19 by the Quantibody Human Inflammation Array-3 (**Figure 1A**). The patients were classified as “severe” during their critical illness or “recovered” based on the conversion to negative nucleic acid test results. Serum samples were collected from these patients at their severe and recovered status, except for patient 4# who was dead from COVID-19 and one serum sample was obtained before the death of the patient (Patient 4# death). We also randomly select a serum sample from a healthy individual (Normal serum). The array result showed strong variations in the cytokine levels among different patients and at different stages for the same patients. Despite these variations, the levels of IL-8 were apparently elevated at the severe stage of all four patients, when compared with recovered and healthy people. Interestingly, the change in IL-6 levels was not so obvious except in patient 4# (**Figure 1B**).

### Cytokine Levels in COVID-19 Severe and Recovered Patients’ Sera

**Figure 2:**
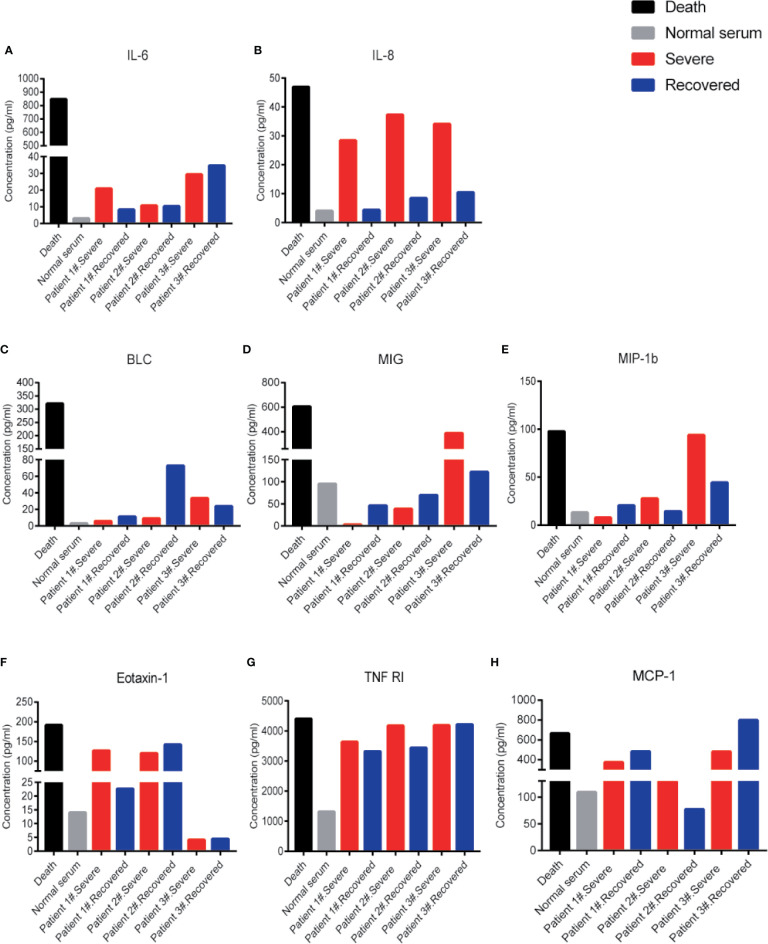
Cytokine levels in COVID-19 severe and recovered patients’ sera. Cytokine levels of IL-6 **(A)**, IL-8 **(B)**, BLC **(C)**, MIG **(D)**, MIP-1b **(E)**, Eotaxin-1 **(F)**, TNF RI **(G)**, and MCP-1**(H)** in COVID-19 severe and recovered patients’ sera. Every column represents one serum sample of COVID-19 patients.

Besides IL-6 and IL-8 levels, other inflammatory cytokines like B-lymphocyte chemoattractant 1 (BLC1, also called CXCL13), monokine induced by IFN-gamma (MIG, also called CXCL9), macrophage inflammatory protein 1 beta (MIP-1b), eotaxin-1 (also called CCL11), tumor necrosis factor-receptor 1 (TNF-R1), and MCP-1 also appeared at very high levels in the patient # 4 compared to the normal serum (**Figures 2A–H**). These cytokines could potentially be indicators of disease severity. Interestingly, the IL-8 levels showed a distinct difference between severe and recovered stages (**Figure 2B**). These results suggest that the level of IL-8 is better than IL-6 as an indicator for COVID-19 disease status.

### IL-8 Performed Better in Distinguishing COVID-19 Patients From Healthy People

**Figure 3:**
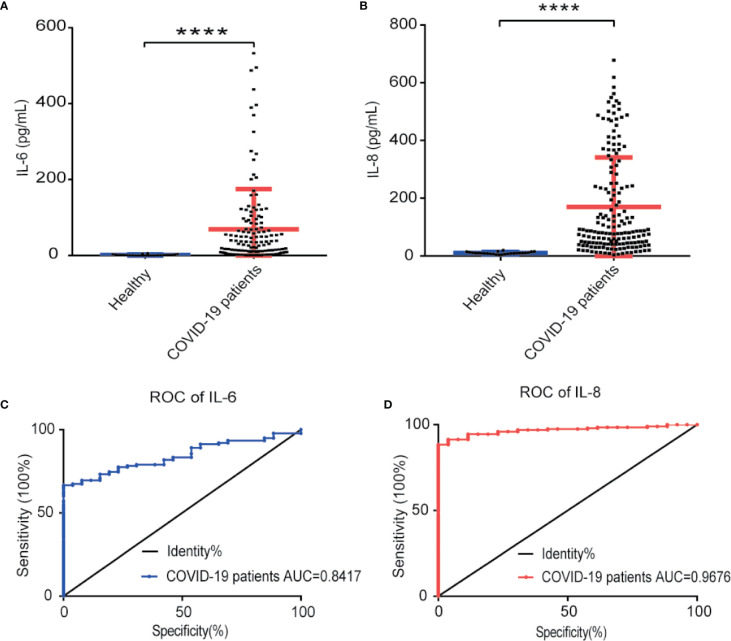
IL-8 performed better in distinguishing COVID-19 patients from healthy people. **(A, B)** Scatter plot of IL-6 **(A)** and IL-8 **(B)** levels detected by ELISA in healthy and COVID-19 patients’ sera. **(C, D)** The detectability performance of IL-6 **(C)** and IL-8 **(D)** in COVID-19 patients was estimated using ROC curve analysis and compared with the AUC. ELISA data **(A, B)** are shown as Mean±SEM, ****p<0.0001, unpaired Student *t* test.

To verify IL-8 as an indicator of COVID-19 disease status, we measured IL-6 and IL-8 concentrations in the sera of 138 COVID-19 patients and 26 healthy people by ELISA assay. Compared to healthy people, both IL-8 and IL-6 levels in the sera of COVID-19 patients were significant higher (**Figures 3A, B**). Receiver operating characteristic (ROC) analysis showed that IL-8 had a higher area under the curve (AUC) (0.9776) than IL-6 (0.8417) (**Figures 3C, D**). The results indicate that IL-8 performs better in distinguishing COVID-19 patients from healthy people.

### While IL-6 Represents for COVID-19 Patients With Severe Disease Conditions, IL-8 Is a Better Indicator of Overall COVID-19 Disease Status

**Figure 4:**
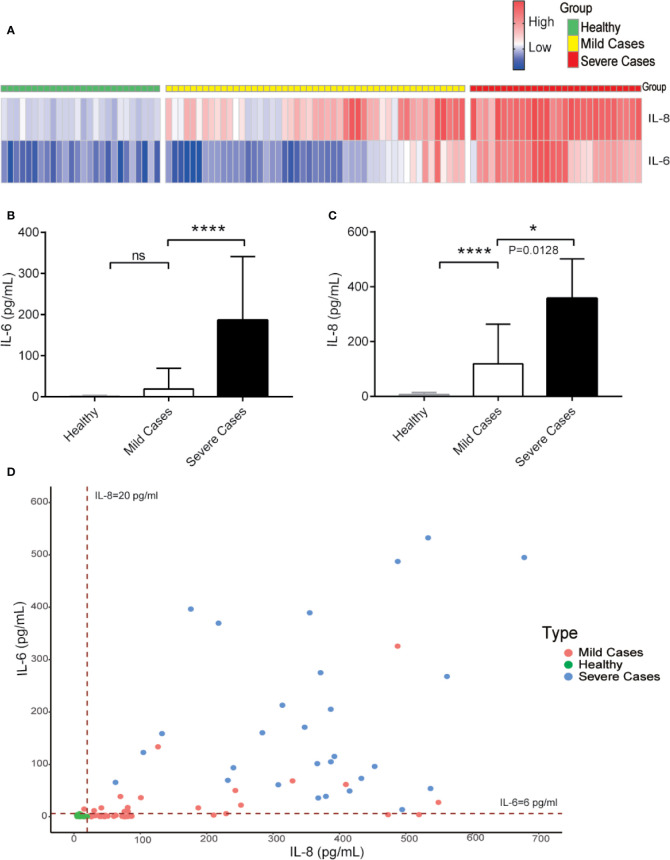
While IL-6 represents for COVID-19 patients with severe disease conditions, IL-8 is a better indicator of overall COVID-19 diseases. **(A)** Heatmap of IL-6 and IL-8 levels from COVID-19 patients during different clinical stages. **(B, C)** The IL-6 **(B)** and IL-8 **(C)** levels among different COVID-19 patient groups. **(D)** Distribution of IL-6 and IL-8 levels among different COVID-19 patient groups. Dashed lines indicate the lower limit of detection for the ELISA. ELISA data **(B, C)** are shown as Mean±SEM, *p<0.05, ****p<0.0001, unpaired Student *t* test.

For the prediction of COVID-19 patient’s disease status, we analyzed IL-6 and IL-8 levels among healthy people, mild, and severe COVID-19 patients. The serum levels of IL-8 were higher in COVID-19 patients with mild symptoms than in healthy people and further elevated in severe COVID-19 patients. However, the serum levels of IL-6 were increased obviously only in severe COVID-19 patients and showed no statistically significant differences between COVID-19 patients with mild symptoms and healthy people (**Figures 4A–D**). These data suggest that while IL-6 represents for COVID-19 patients with severe disease conditions, IL-8 is a better indicator of overall COVID-19 disease status.

### IL-8 May Serve as a Biomarker to Indicate the COVID-19 Disease Prognosis

**Figure 5:**
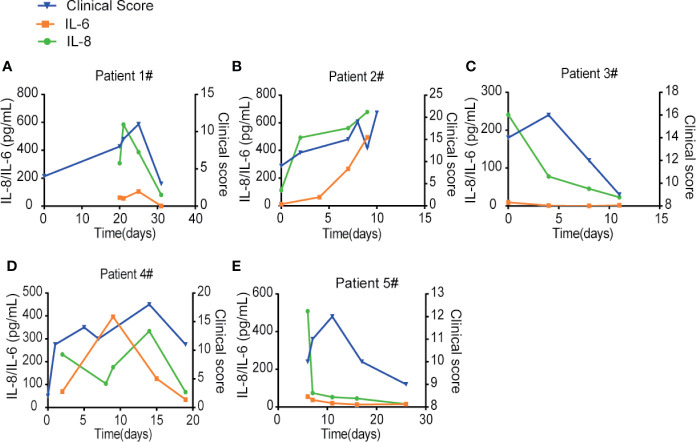
IL-8 may serve as a biomarker to indicate the COVID-19 disease prognosis. **(A–E)** IL-6 and IL-8 levels were compared with different patients’ clinical scores at multiple time points. Left Y-axis displays the concentration of cytokines: IL-6 in orange and IL-8 in green. Right Y-axis displays the clinical scores depicted in blue. X-axis displays the day numbers of COVID-19 diagnosis from the first positive PCR test.

To compare IL-6 versus IL-8 for the disease prognosis prediction, we monitored the changes of IL-6 and IL-8 concentrations in the disease progression of five COVID-19 patients with different clinical scores, which represent a combination of multiple physiologic measurements associated with the COVID-19 diseases. The levels of IL-8 seemed to correlate well with different patient’s clinical scores at multiple time points (**Figures 5A–E**). On the other hand, the levels of IL-6 remained low at most of the time points except patients at very high clinical scores. Our studies therefore indicate that IL-8 may serve as a biomarker to indicate the COVID-19 disease prognosis.

## Discussion

Since the outbreak of SARS-CoV-2 infection, the global pandemic of COVID-19 has caused about a million deaths among over fifty million confirmed cases worldwide (34–36). Although PCR-based detection of SARS-CoV-2 viral RNA and ELISA-based detection of antibodies against SARS-CoV-2 viral proteins have been developed for the diagnosis of infected versus uninfected patients (37, 38), there is urgent need to identify biomarkers for the disease prognosis of COVID-19 patients. In the present studies, we have provided evidence that while IL-6 may be used for the diagnosis of severe COVID-19 patients, IL-8 is overall a better biomarker for the COVID-19 disease prognosis.

SARS-CoV-2 infection caused a wild range of symptoms among different COVID-19 patients. The outcomes of COVID-19 patients also varied from recovery to death. While young and healthy COVID-19 patients may be asymptomatic or have mild symptoms, elderly patients and those with preexisting health conditions tend to develop severe and fatal disease after infection with SARS-CoV-2 (5, 39). Because of the wide and variable range of clinical symptoms and progression, it is difficult to predict the disease progress and outcome for individual COVID-19 patients. Through comparing the cytokine levels in the serum samples of COVID-19 patients at longitudinal timepoints during severe illness or at recovery, we have identified differential cytokine profiles potentially associated with COVID-19 disease status. Besides IL-6, which has been reported as a potential biomarker for COVID-19 patients (28, 40), we found that both IL-6 and IL-8 serum levels were elevated in COVID-19 patients with severe diseases. In particular, we found that serum levels of IL-6 and IL-8 were significantly higher in COVID-19 patients as compared with heath donors with the AUC, which represents the combination of detecting sensitivity and specificity, at 0.84 and 0.98, respectively. More importantly, our studies indicated that IL-6 and IL-8 may be differentially used as biomarkers for the severity and prognosis of the COVID-19 associated diseases. The levels of IL-6 were low in most of the COVID-19 patients with mild symptoms and were elevated in patients with severe symptoms, which therefore may be used as a biomarker for severe COVID-19 patients (25, 41). On the other hand, the serum levels of IL-8 were remarkably higher in COVID-19 patients with either mild or severe diseases as compared with the healthy donors. Furthermore, the IL-8 levels correlated better than IL-6 with the overall clinical disease scores at the different time points in the same COVID-19 patients, which can therefore be used as a disease prognosis biomarker.

Cytokine storms have been reported as a part of the COVID-19 associated severe diseases (24, 42, 43). Blocking antibodies or small molecules targeting IL-6 or its signal transduction pathways have been used in the clinical trials to treat COVID-19 patients (23, 29). However, targeting IL-6 signal transduction pathways might be complicated as IL-6 activates both pro-inflammatory pathway through the soluble form of the IL-6 receptor, and anti-inflammatory pathway through the membrane-bound form of the IL-6 receptor (27, 44). IL-8 is clearly a pro-inflammatory cytokine that may recruit neutrophils to the infected areas and has been associated with tissue damage. Our studies therefore suggest the possibility of using IL-8 as a biomarker for the disease prognosis of COVID-19 patients and as a potential therapeutic target to treat COVID-19 patients.

## Conclusion

Although IL-6 is indicated as a biomarker in predicting severe status of COVID-19 patients, IL-8 is an easily detectible and sensitive biomarker in either mild or severe COVID-19 patients. Serum levels of IL-8 correlated better than IL-6 levels with the overall clinical disease scores at different stages of the same COVID-19 patients. Thus, IL-8 may act as a biomarker for COVID-19 disease prognosis and target for COVID-19 treatment in the future.

## Data Availability Statement

The original contributions presented in the study are included in the article/supplementary material. Further inquiries can be directed to the corresponding authors.

## Ethics Statement

The studies involving human participants were reviewed and approved by The Ethics Committee of Taihe hospital (2020KS031). The patients/participants provided their written informed consent to participate in this study. The animal study was reviewed and approved by The Ethics Committee of Taihe hospital (2020KS031). Written informed consent was obtained from the individual(s) for the publication of any potentially identifiable images or data included in this article.

## Author Contributions

GC and HY jointly designed this study. YW, JiL, XL, JZ, XX, YJ, and CC performed Quantibody human inflammation array and ELISA assay. LL, MG, MS, and HF collected patient serum samples and clinical data analysis. GC, HY, LL, FM, JK, and SA wrote and revised the manuscript. All authors contributed to the article and approved the submitted version.

## Funding

This project is supported by the Research Funds from Chinese Academy of Medical Sciences (2016-I2M-1-005 and 2019XK310002), National Science funds (2015ZX09102023, NSFC 91542201, 81590765, 31670883, 31870912, 81802870, and 2017YFA0506200), US National Institute of Health funds (AI069120, AI056154, AI140718, and AI028697), the UCLA AIDS Institute and UCLA David Geffen School of Medicine – Eli and Edythe Broad Center of Regenerative Medicine and Stem Cell Research Award Program. HY is supported by Non-profit Central Research Institute Fund of Chinese Academy of Medical Sciences (3332018131), Science funds from Jiangsu Province (BK20170407, BK20151253) and The Innovative and Entrepreneurial Team grant (2018-2021) from Jiangsu Province. LL is supported by Chinese Postdoctoral Science Foundation (2019M650564), CAMS Innovation Fund for Medical Sciences (CIFMS; 2019-I2M-1-003) and Innovative and Entrepreneurial Doctor grant (2020-2022) from Jiangsu Province. JiL is supported by Natural Science Foundation of Hubei Province Grant (2016CFB154) and the Scientific and Technological Project of Shiyan City of Hubei Province (18Y26). MS is supported by Key Project of Jiangsu Provincial Health Commission (NO. K2019021). 

## Conflict of Interest

Authors YW and CC were employed by the company Suzhou Func Biotech Inc.

The remaining authors declare that the research was conducted in the absence of any commercial or financial relationships that could be construed as a potential conflict of interest.
